# Cell Biological Characterization of the Malaria Vaccine Candidate Trophozoite Exported Protein 1

**DOI:** 10.1371/journal.pone.0046112

**Published:** 2012-10-08

**Authors:** Caroline Kulangara, Samuel Luedin, Olivier Dietz, Sebastian Rusch, Geraldine Frank, Dania Mueller, Mirjam Moser, Andrey V. Kajava, Giampietro Corradin, Hans-Peter Beck, Ingrid Felger

**Affiliations:** 1 Medical Parasitology and Infection Biology, Swiss Tropical and Public Health Institute, Basel, Switzerland; 2 University of Basel, Basel, Switzerland; 3 Department of Biochemistry, University of Lausanne, Epalinges, Switzerland; 4 Centre de Recherches de Biochimie Macromoleculaire, Centre national de la recherche scientifique, University of Montpellier 1 and 2, Montpellier, France; Universidade Federal de Minas Gerais, Brazil

## Abstract

In a genome-wide screen for alpha-helical coiled coil motifs aiming at structurally defined vaccine candidates we identified PFF0165c. This protein is exported in the trophozoite stage and was named accordingly Trophozoite exported protein 1 (Tex1). In an extensive preclinical evaluation of its coiled coil peptides Tex1 was identified as promising novel malaria vaccine candidate providing the rational for a comprehensive cell biological characterization of Tex1. Antibodies generated against an intrinsically unstructured N-terminal region of Tex1 and against a coiled coil domain were used to investigate cytological localization, solubility and expression profile. Co-localization experiments revealed that Tex1 is exported across the parasitophorous vacuole membrane and located to Maurer's clefts. Change in location is accompanied by a change in solubility: from a soluble state within the parasite to a membrane-associated state after export to Maurer's clefts. No classical export motifs such as PEXEL, signal sequence/anchor or transmembrane domain was identified for Tex1.

## Introduction

In the past few years Tex1 encoded by PFF0165c was characterized as a novel malaria vaccine candidate. According to PlasmoDB version 6.5 (http://plasmodb.org) *tex1* spans nucleotide positions 133′147 to 136′458 on chromosome 6. Tex1 had been identified originally in a genome-wide screen of alpha-helical coiled coil domains in a search for novel vaccine candidates against the blood stage of *P. falciparum* [1], [2]. Chemically synthesized short peptides consisting of such a motif can fold into their native structure in aqueous environment and therefore mimic structurally native epitopes. Two regions of Tex1 were chemically synthesized. One of the synthetic peptides, P27, is spanning the coiled coil domain (K845 to T871), the other, P27A, corresponds to N-terminal intrinsically unstructured region (H223 to S326). Both peptides were tested in an extensive preclinical evaluation protocol to analyze the properties of anti-P27 and anti-P27A antibodies regarding *in vitro* parasite killing in presence of monocytes [1], [3], correlation with protection in adults and children [3], [4], prevalence of peptide recognition by sera from semi-immune adults from different endemic region throughout the world [1], [3] and sequence conservation in different culture strains and field isolates [3], [5]. Both fragments of Tex1, peptides P27A and P27, are considered promising novel malaria blood stage vaccine candidates. A phase 1 clinical study of P27A is scheduled in 2011.

In view of the promising outcome of preclinical evaluation and the imminent phase 1 clinical trial, a comprehensive biological characterization of Tex1 was called for. Here we present results of a cell biological analysis characterizing Tex1 in relation to other known exported parasite proteins. We show that Tex1 associates to Maurer's clefts (MC) membrane facing the cytosol of the RBC. Tex1 export depends on the classical secretory pathway. But it seems to lack a classical signal sequence as well as a PEXEL motif, suggesting the presence of alternative sequences involved in protein export to the PV and across the PVM to the RBC cytosol.

## Materials and Methods

### Ethical treatment of animals

The animal work has been carried out according to relevant national and international guidelines. The immunization experiments in CB6F1 mice and the immunization protocol was approved by the Canton de Vaud (Permit number: 805.6). Immunization of rabbits were performed by the commercial company Eurogentec, 4102 Seraing, Belgium.

### Cell culture and protein extracts

*P. falciparum* 3D7 strain was cultured at 5% haematocrit as described [6], using RPMI medium supplemented with 0.5% Albumax [7]. Parasites were synchronized with 5% sorbitol [8]. To obtain protein extract of mixed stage infected erythrocytes parasites (10 ml petri-dish) were grown to 5% to 10% parasitemia, lysed on ice in 0.03% saponin in phosphate-buffered saline (PBS, pH 7.4) for 10 min, washed with ice cold PBS for complete removal of hemoglobin, and resuspended in Laemmli sample buffer. The protein extracts of late-stage parasites (trophozoites and schizonts) were obtained from *P. falciparum* 3D7-infected erythrocytes in a 30-ml petri dish (5% hematocrit, 6% parasitemia) which was enriched using a magnetic cell sorter (Miltenyi Biotec, Bergisch Gladbach, Germany). The enriched infected erythrocytes were lysed in a 200 µl volume of PBS, 0.03% saponin (Fluka) in the presence of protease inhibitors (Roche Diagnostics, Basel, Switzerland) for 5 min at 4°C. The parasites were pelleted by centrifugation at 4,000×*g* for 10 min, the supernatant was collected and mixed with sample buffer. The parasite pellet was resuspended in 0.1 M Tris, pH 6.8, and an equal volume of 2× Laemmli sample buffer. For protein expression profiling 5 ml of tightly synchronized culture (2 h time frame; 8% parasitemia) was harvested in a 4 hours interval, parasites were lysed on ice in 0.03% saponin in PBS for 10 min and wash 3 times in ice-cold PBS. Parasite pellet was resuspended in cold 0.1 M Tris, pH 6.8, and an equal volume of 2× Laemmli sample buffer.

### Recombinant expression and purification of recP27 fragment

**Figure 1:**
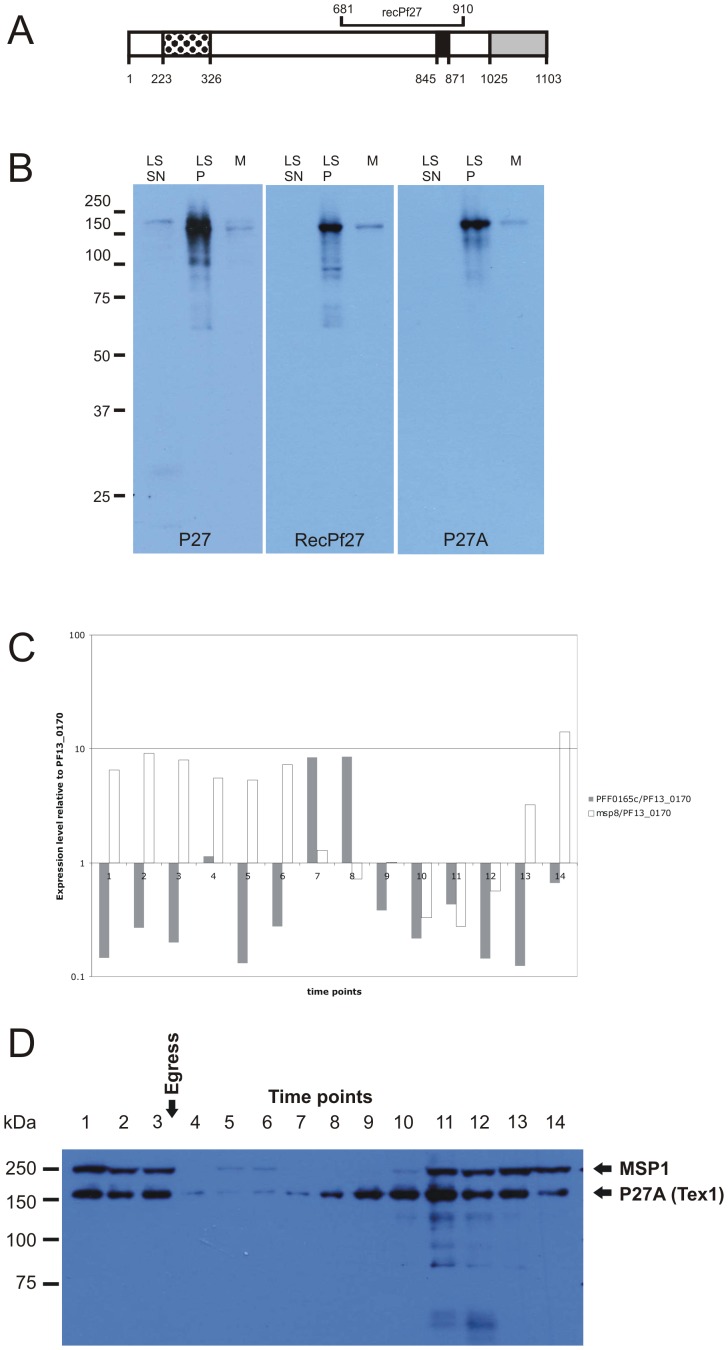
Tex1 structure and expression dynamics on transcriptional and protein level. A) Schematic representation of Tex1. Black dotted: intrinsically unstructured region P27A; Black: coiled coil domain P27; grey: RING motif. B) Western Blot analysis of antibodies specific for P27A and P27 on the pellet fraction after saponin lysis of mixed stage parasite (M) and late stage parasite (LS). The late stage parasites were fractionated into a supernatant (SN) and a pellet (P) fraction after saponin lysis. M: marker C) Abundance of *tex1* transcripts by gRT-PCR. RNA was isolated from tightly synchronized culture in a 4h interval (table 1). *Tex1* transcript levels (grey bars) were normalized to the transcript abundance of the constitutively expressed glutaminyl-tRNA synthetase (PF13_0170). As a control for the level of synchronization *msp8* transcripts were measured and compared to the PF13_0170 transcript level (white bars). D) Protein level was analyzed throughout the intraerythrocytic development cycle in a 4 h interval by Western Blot analysis using antibodies against P27A and compared to protein abundance of MSP1. The parasite age (in hours post infection) and the parasite stages (confirmed by Giemsa staining) corresponding to the time points of harvest are illustrated in table 1.

The C-terminal fragment of Tex1 containing the coiled coil motif P27 (Figure 1A, M681 to E910) was amplified from 3D7 genomic DNA by PCR and cloned into the pQE60 plasmid via the NcoI and BamHI restriction sites (primers used are listed in **Table S1**). Recombinant expression was performed following the manufacturer's protocol (Qiagen Inc.).

### Generation of anti-P27, anti-P27A and anti-recPf27 polyclonal rabbit sera and anti-P27 polyclonal mouse sera

Rabbit sera were produced by Eurogentec, Seraing, Belgium. In short, the recPf27 protein (250 µg) was used for immunization with Freud's adjuvant into two New Zealand white rabbits. Sera samples (20 ml) were affinity purified using recPf27-6xHis protein or the P27 coiled coil peptide coupled to HiTrap NHS-activated HP columns (GE-Healthcare, 1 ml). After antibody binding columns were washed with 50 ml PBS, bound IgG was eluted with 0.1 M glycine, pH 2.5, and the buffer was subsequently changed to PBS using HiTrap Desalting Columns (GE Healthcare). Purified antibodies were stored at −80°C until further use. Polyclonal mouse sera was obtained by immunization of CB6F1 mice. CB6F1 mice were injected 3 times with 20 µg of the P27 peptide in Montanide ISA 720 at the base of the tail on day 1, 22 and 78. Bleeding was performed 10 days after the second and third immunization. Affinity purification of P27A-specific rabbit has previously been described [1], [3].

### Western blot analysis

Protein extracts were separated on a 10% sodium dodecyl sulfate-polyacrylamide gel and transferred to nitrocellulose (Hybond-C extra; GE Healthcare) at 4°C for 1 h at 80 V and an additional hour at 100 V. The membrane was blocked for 1 h in 5% skim milk, 0.1% Tween in Tris-buffer. Antibodies used were: Polyclonal rabbit anti-P27A (1∶5000); anti-P27 (1∶2500) and anti-Pf27rec (1∶2500); anti-MAHRP1 (1∶5000), monoclonal mouse anti-MSP1 ([9], 1∶1000); anti-SERA5 ([10], 1∶2000); horseradish peroxidase-conjugated goat anti-mouse (Pierce, 1∶20 000), goat anti-rabbit (Acris, 1∶10000).

### Solubility analysis

*P. falciparum* 3D7-infected erythrocytes (30-ml petri dish; 6% parasitemia) were enriched using a magnetic cell sorter (Miltenyi Biotec, Bergisch Gladbach, Germany). Purified mature stages were resuspended in 200 µl 5 mM Tris pH 8 in the presence of protease inhibitors (Roche Diagnostics, Basel, Switzerland) and lysed by 3 freezing-thawing cycles. Soluble protein fraction was separated by centrifugation 30 min at 20 000×g at 4°C. The membrane-containing pellet was resuspended in 200 µl 0.1 M Na_2_CO_3_ and incubated for 30 min on ice to extract peripheral membrane proteins. Supernatant containing peripheral proteins was separated by centrifugation (30 min at 20 000×g at 4°C). Integral membrane proteins were extracted from the pellet with 1% Triton X-100 on ice for 30 min. Supernatant containing integral proteins was separated by centrifugation (30 min at 20 000×g at 4°C) The remaining proteins were extracted with 4% SDS, 0.5% TritonX-114 in 0.5× PBS for 30 min at room temperature and separated from the pellet by centrifugation. The supernatant was analyzed as insoluble protein fraction. 10 µl of each fraction was analyzed by Western Blot.

### RNA isolation, cDNA synthesis and real time PCR

3D7-infected erythrocytes were tightly synchronized using 5% D-sorbitol [8]. Three rounds of 5% D-sorbitol treatment was applied (2^nd^ and 3^rd^ treatment was applied 8 hours and 14 hours after the 1^st^ D-sorbitol treatment). Parasites were grown to 8–10% parasitemia (5% heamatocrit). 1.5 ml culture was taken in 4 h intervals. In brief, parasite RNA was extracted using TRIzol (Invitrogen) according to the manufacturer's instructions. TRIzol extraction was repeated. Residual gDNA was digested twice with RQ1 DNase (Promega) according to the manufacturer's protocol. Reverse transcription was done by AffinityScript Multiple Temperature Reverse Transcriptase (Stratagene) with random primers (Invitrogen) as described by the manufacturer. To control for gDNA contamination, the target sequence was amplified from the RNA solution prior to reverse transcription. Absolute transcript quantification was performed at final primer concentrations of 0.4 µM using SYBR® Green Master Mix (Applied Biosystems) on a StepOnePlus™ Real-Time PCR System (Applied Biosystems) in a reaction volume of 12 µl. All reactions were performed in triplicate yielding virtually identical Ct values. A serial dilution of gDNA was used as standard for absolute quantification. Relative transcript profiles were calculated by normalization against transcript levels of the house-keeping gene PF13_0170 (glutaminyl-tRNA synthetase). Validation of synchronization procedure was obtained by analyzing transcript levels of the merozoite surface protein 8 (*msp8*). The primers used for qPCR of *tex1*, PF13_0170 and *msp8* are shown in **Table S2**. The time points of harvest (1–14), the corresponding age of parasites (in hours post infection) and the corresponding parasite stages are illustrated in **Table 1** and were confirmed by Giemsa staining before RNA and Protein extraction.

**Table 1:** Time points of harvest of synchronized 3D7 *in vitro* culture.

Time points	Hours post infection	Parasite stage
1	46–48	Schizont/Ring
2	0–2	Schizont/Ring
3	4–6	
4	8–10	Ring
5	12–14	
6	16–18	Late Ring
7	20–22	Early Trophozoite
8	24–26	
9	28–30	Trophozoite
10	32–34	
11	36–38	Late Trophozoite
12	40–42	Late Trophozoite/Schizont
13	44–46	Schizont
14	48–50	Schizont/Ring

Synchronized *P. falciparum* 3D7 parasite culture were harvested in 4 hours interval. Time points of harvest 1–14 (column 1), the corresponding age of synchronized parasites at each time point of harvest (in hours post invasion, column 2) and the corresponding parasite stage (column 3).

### Indirect immunofluorescence assay (IFA)

Infected erythrocytes were fixed with 4% paraformaldehyde (Polyscience) and 0.0075% glutaraldehyde (Polyscience) for 30 min under constant agitation, permeabilized using 0.1% Triton X-100/PBS for 10 min and blocked with sodium borohydride (NaBH_4_)/PBS for 10 min followed by an additional blocking step using 3% BSA in PBS for 1 h as described in [11]. Primary antibodies were used with the following concentrations: polyclonal rabbit sera anti-P27A (1∶2000); anti-P27 (1∶1000), anti-MAHRP2 ([12], 1∶100); anti-REX1 (kind gift from Prof. Don Gardiner, 1∶500); mouse polyclonal antibodies anti-P27 (1∶200); anti-SBP1 N-terminus specific (kind gift from Prof. Catherine Braun-Breton, 1∶200); anti-MAHRP1 (1∶200). Secondary antibodies used: Alexa Fluor 488 (Invitrogen; 1∶400); Texas Red (Invitrogen; 1∶400). Cells were mounted in Vectashield Hard Set supplemented with DAPI (Vector Laboratories) for staining of the nuclear DNA. For the Equinatoxin II assay infected erythrocytes were lightly fixed with 2% paraformaldehyde in RPMI medium (10 min), permeabilized with Equinatoxin II [13], and re-fixed with 4% formaldehyde and 0.00075% glutaraldehyde in PBS (pH 7.4, Gibbco). Cells were blocked with 3% BSA (Sigma) in PBS. Cells were split and one half was additionally treated with 0.1% triton (Merck) for complete permeabilization. Synchronized Ring stage parasites (aged 4 h to 8 h post invasion) were treated with Brefeldin A solved in 100% ethanol (Fluka) to a final concentration of 5 µg ml^−1^. Control cultures were incubated in the presence of equivalent amounts of ethanol. After 18 h parasites were fixed for IFA. BFA was removed from the remaining parasites which were further cultured to ensure viability after treatment. Images were obtained by the Zeiss confocal microscope LSM 700 or Leica DM 5000B fluorescence microscope. Images were processed by ImageJ software or the Huygens Essential Software (Scientific Volume Imaging, The Netherlands). Quantitative analysis of co-localization was done with Huygens Essential Software (Scientific Volume Imaging, The Netherlands).

## Results

According to PlasmoDB version 6.5 (http://plasmodb.org), the predicted protein has a length of 1103 amino acids (aa) and contains 3 predicted coiled coil domains. Alpha-helical coiled coils share the heptad motif (**abcdefg**)_n_ with positions **a** and **d** representing hydrophobic residues, whereas the remaining positions are generally polar. Depending on slight variations in their sequences the coiled coil bundles consist of 2 to 7 alpha-helices that spontaneously self assemble in aqueous solutions. One of the 3 coiled coil domains in Tex1 is the P27 region from position K845 to T871 (in black, **Figure 1A**.), which has been identified as potential malaria vaccine candidate previously [1]. The C-terminus of Tex1 consists of a predicted RING (Really Interesting New Gene) domain, spanning amino acids K1025 to L1102 (in grey, **Figure 1A**). Furthermore, a large portion of the C-terminal half of this protein (650–1040) has sequence similarity to several proteins with known 3D structure which have elongated alpha-helical domains capped by the RING-domains (e.g. [14]. This supports correctness of our previous prediction of alpha-helical coiled coil regions in Tex1 (Villard et al., 2007). A long intrinsically unstructured region (IUR) named P27A, ranging from position H223 to S326 (black dotted, **Figure 1A**), corresponds to the second identified potential vaccine candidate within Tex1 [3]. P27A is currently under clinical development and a phase 1 clinical trial is scheduled for 2011.

### Tex1 is expressed in intraerythrocytic blood stage parasites and its transcription is up-regulated in the early trophozoite stage

In order to characterize the protein by IFA and Western Blot, rabbit antibodies were generated against P27A and a 240 aa long (including linker and His-tag) recombinant protein (recPf27) encompassing amino acid M681 to E910 (**Figure 1A**) in the C-terminal part of Tex1 and including the P27 coiled coil domain (K845 to T871). Both polyclonal rabbit sera were affinity purified on the respective immunogens. In addition, recPf27 rabbit serum was alternatively affinity purified on the P27 peptide. Thus, three polyclonal rabbit sera were available with specificities to P27A, recPf27 and P27.

Previously, we showed that P27A specific mouse and rabbit sera both detected a protein with the mass of 160 kDa in Western Blot broadly consistent with the predicted mass of 132 kDa [3]. When using several sera raised against different parts of Tex1, all sera recognized a band at about 160 kDa both, in the pellet fraction of mixed parasite stages and in late stage parasites (**Figure 1B**).

The transcription profile was analyzed by quantitative real-time PCR on RNA from tightly synchronized cultures harvested in 4 h intervals covering the 48 h intra-erythrocytic developmental cycle. The collected time points, the corresponding age of the parasites (in hours post invasion) and the respective parasite stages are listed in table 1. The transcription level of *tex1* was analyzed in relation to that of a constitutively transcribed gene, *glutaminyl-tRNA synthetase* (PF13_0170). *Tex1*-specific transcripts were detected throughout the intra-erythrocytic development cycle, but an up-regulation of transcript abundance was detected in early trophozoites (gray bars, **Figure 1C**). To validate the synchronization procedure, transcript levels of the merozoite surface protein 8 (*msp8*) were analyzed at each time point. The *msp8* profile obtained (white bars, **Figure 1C**) showed an up-regulation in ring stages and in very late schizont stages as shown in PlasmoDB. The RNA levels of *tex1* were compared with a time course of Tex1 protein abundance analyzed by Western Blot during the intra-erythrocytic cycle. Tex1 protein levels detected in 4 h intervals were highest during early trophozoite stage at time point 8 (**Figure 1D**). The protein persisted until egress, reflected. in the presence of the full length merozoite surface protein 1 (MSP1) [15].

### Tex1 is exported to the host cell cytosol and localizes to Maurer's clefts

**Figure 2:**
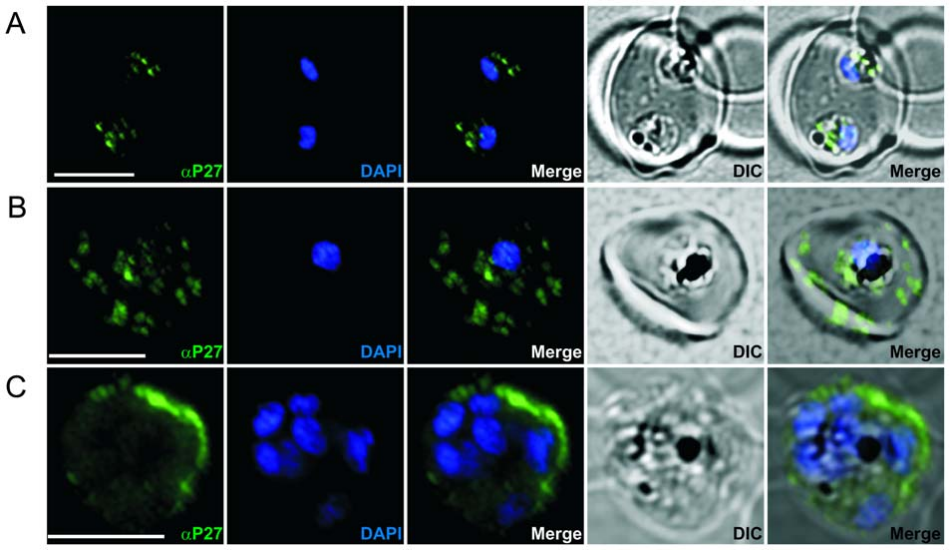
Immunofluorescence staining of erythrocytes infected by *P. falciparum* (ring, trophozoites and schizont stages) using P27-specific polyclonal rabbit sera. P27-specific polyclonal rabbit sera was used to detect Tex1 (green) A) in late ring stages B) in trophozoite stages C) in schizont stages. Nucleus stained with DAPI (blue), transmission picture of the infected red blood cell (DIC) and merged picture of the two signals or the signals merged with transmission picture (merge), Scale bar: 5 µm.

Previously we reported that Tex1 was exported and accumulated at structures in the cytosol of the infected RBC [3]. To study the exact subcellular localization of Tex1 during the intra-erythrocytic cycle, synchronized 3D7 parasites were analyzed by IFA. In early ring stages (0–6 hours post invasion) the protein was absent (data not shown), whereas in late ring stages (12–16 hours post invasion) Tex1 was detected in punctuated structures within the parasite (**Figure 2A**). In trophozoite stages, Tex1 is exported to the host cell cytosol and associates with elongated structures in the cytosol of the infected RBC (**Figure 2B**) suggestive of Maurer's clefts (MC) staining [16]. In schizont stages the protein was much less focused and seemed to associate to the periphery of the host cell in vicinity to the host cell membrane (**Figure 2C**).

**Figure 3:**
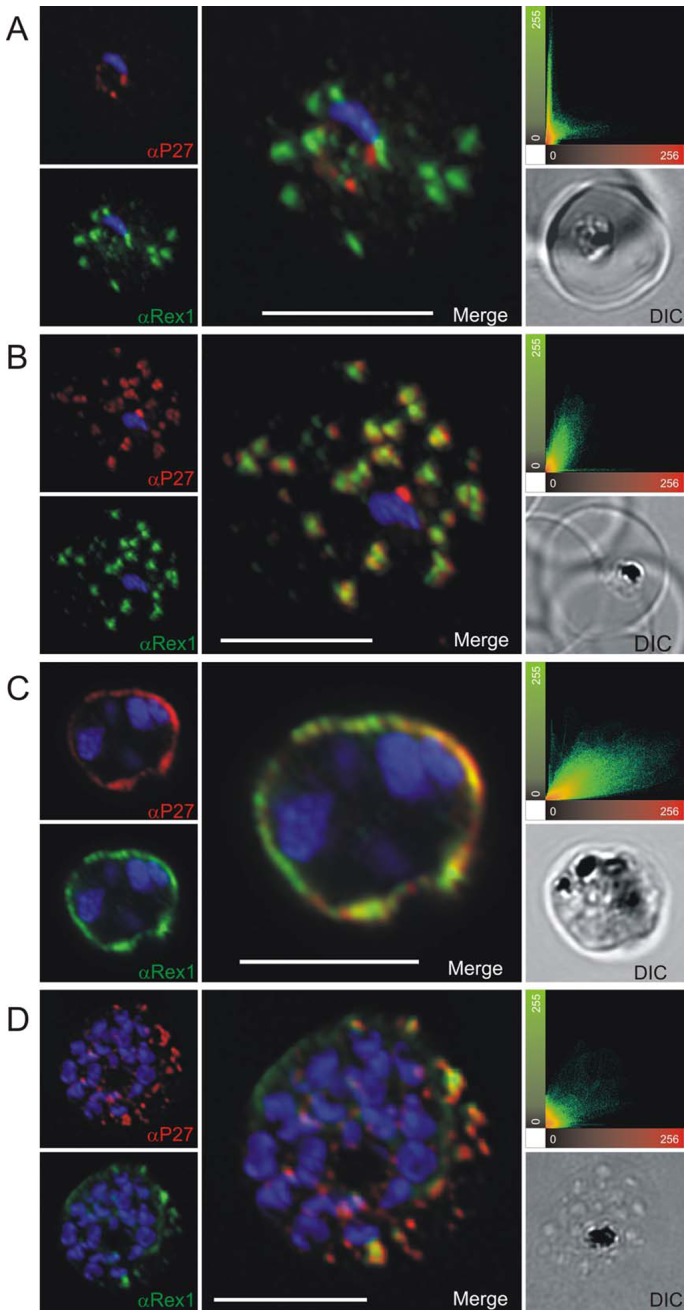
Co-localization of Tex1 with Rex1. P27-specific polyclonal mouse sera (in red) was used to detect TEX1. Rex1 polyclonal rabbit sera (in green). (A) Ring stage parasites; (B) trophozoite stages; (C) schizont stages. Scatter plots show the degree of co-localization of the Tex1 with Rex1 signal. Nuclear DNA was stained with DAPI (blue), Transmission image (DIC), Scale bar: 5 µm.

**Figure 4:**
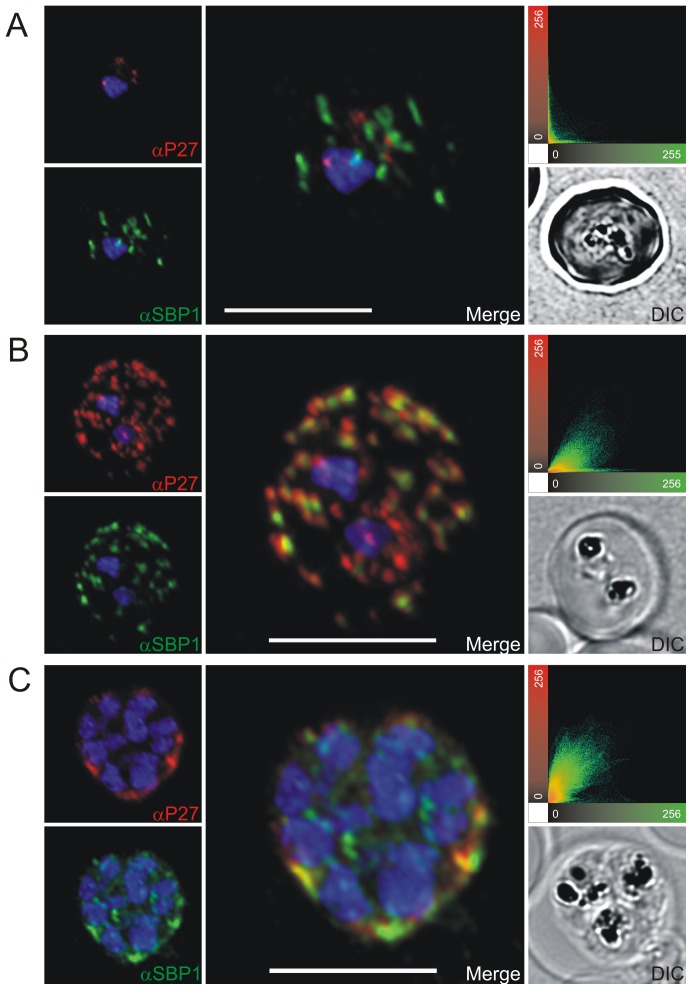
Co-localization of Tex1 with SBP1. P27-specific polyclonal rabbit sera was used to detect Tex1 (red). Co-localization was performed using SBP1 polyclonal mouse sera (green). Co-localization was performed in ring (A) trophozoite (B) and schizont stage (C) infected RBCs. Nuclear DNA was stained with DAPI (blue), Transmission image (DIC), Scale bar: 5 µm.

**Figure 5:**
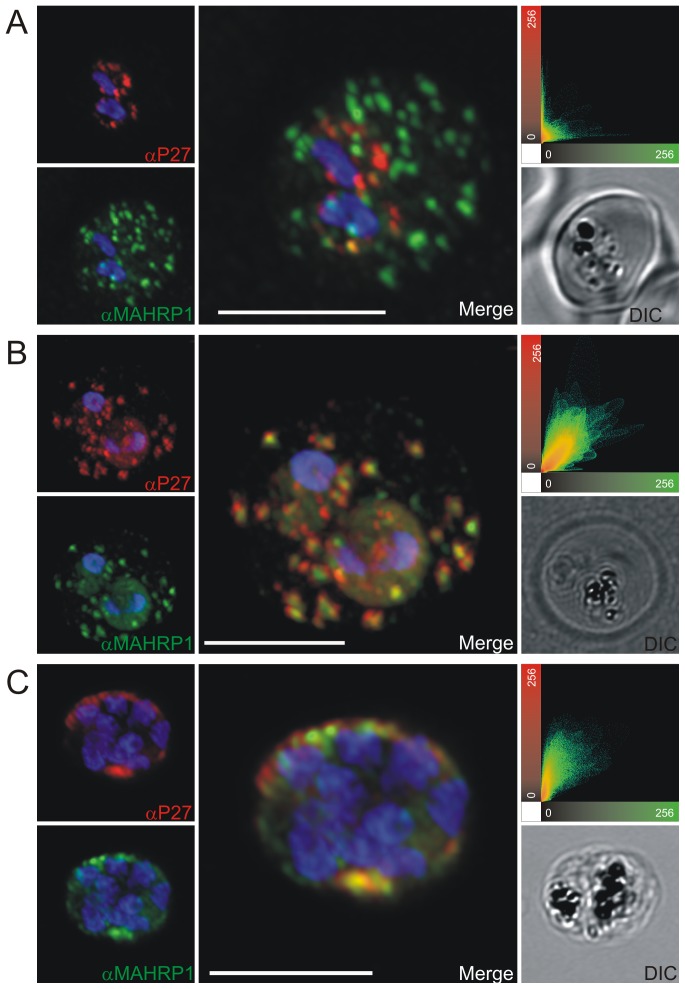
Co-localization of Tex1 with MAHRP1. P27-specific polyclonal rabbit sera was used to detect Tex1 (red). Co-localization was performed using MAHRP1 polyclonal mouse sera (green). Co-localization was performed in ring stage (A) trophozoite (B) and schizont stage (C) infected RBC. Nuclear DNA was stained with DAPI (blue), Transmission image (DIC), Scale bar: 5 µm.

**Figure 6:**
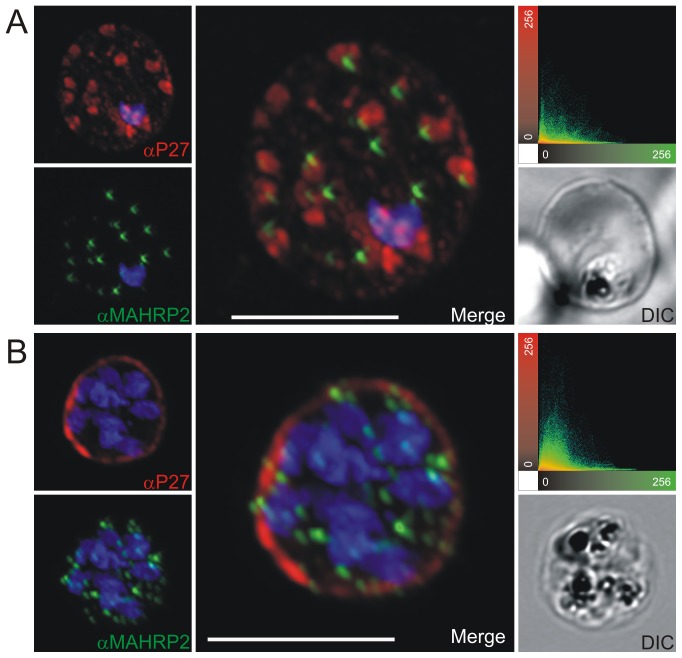
Tex1 localization in trophozoite and schizont stages with respect to newly described structures called tethers. Co-localization of Tex1 (red) with MAHRP2 (green) A) in trophozoite stages and B) in schizont stages. Nuclear DNA was stained with DAPI (blue), Transmission image (DIC), Scale bar: 5 µm.

To prove the localization to MC, co-localization experiments were performed using antibodies against known MC markers. In late ring stages the ring exported protein 1 (Rex1) (**Figure 3A**), SBP1 (**Figure 4A**) and MAHRP1 (**Figure 5A**) associated with MC, whereas Tex1 still remained within the parasite. In trophozoite, schizont and late schizont stages, Tex1 appeared to associate with MC as demonstrated by co-localization with Rex1 (**Figure 3B, 3C, 3D**), SBP1 (**Figure 4B, 4C**) and MAHRP1 (**Figure 5B and 5C**. In schizont stages Tex1 signal was detected similar to Rex1 adjacent to the RBC membrane. Tex1 was also detected in close proximity to new structures called tethers (**Figure 6A**) that are characterized by the membrane-associated histidine rich protein 2 (MAHRP2, [12]. However, Tex1 is not found anymore in close proximity to MAHRP2 in schizont stage parasites (**Figure 6B**). Antibodies directed against Tex1 failed to detect the protein at the surface of infected RBCs in unpermeabilized cells (**Figure S1**) suggesting that in schizonts the protein resides inside of the infected cell in close proximity to the RBC membrane.

### Tex1 occurs in two conditions: as soluble protein and in association with membrane structures

**Figure 7:**
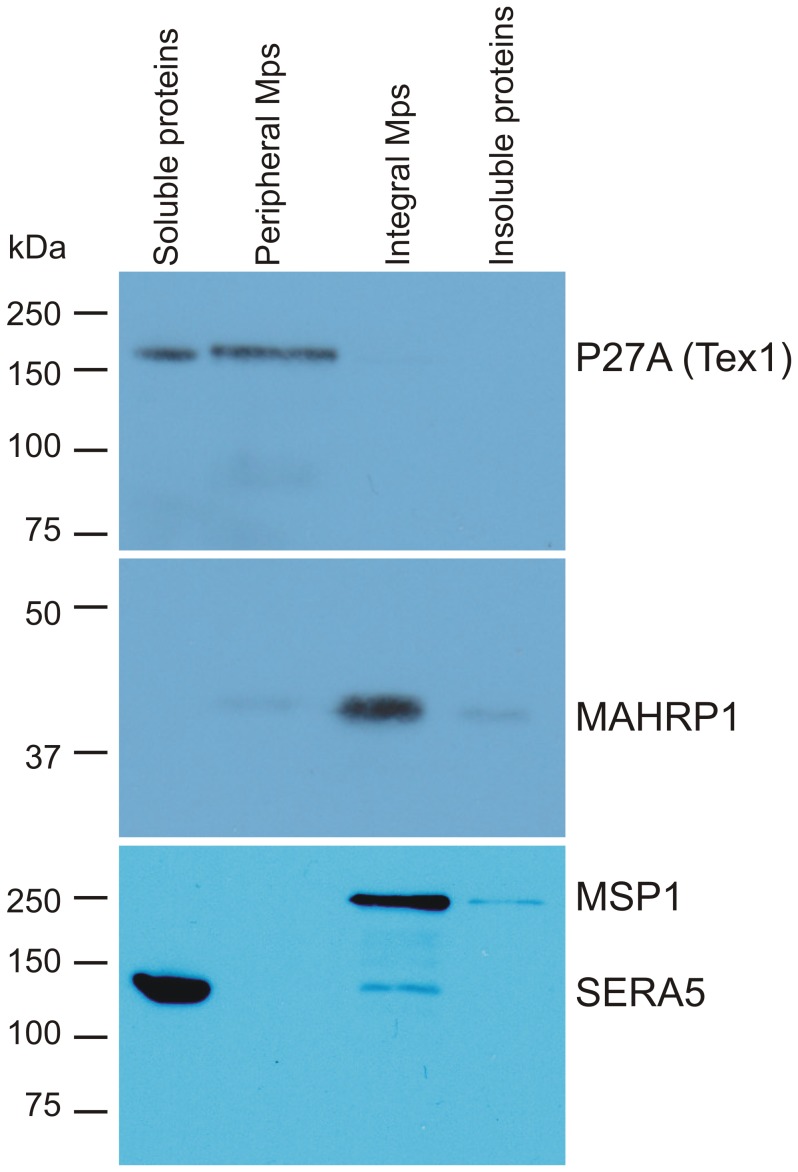
Dual solubility pattern of Tex1 shown by Western blot analysis of membrane fractionation assay of late stage parasites. Soluble proteins from membranes of RBCs infected with late stage parasites lysed by freezing thawing cycles (lane 1). Peripheral membrane proteins extracted by sodium carbonate buffer, (lane 2). Integral membrane proteins obtained by additional 1% Triton X-100 extraction (lane 3). Insoluble proteins (remaining membrane proteins after Triton X-100 extraction (lane 4). Blot was probed with P27A-specific polyclonal rabbit sera (panel 1), anti-MAHRP1 polyclonal rabbit sera (panel 2) and SERA5 and MSP1 mouse monoclonal antibodies (panel 3).

Late parasite stages were purified by magnetic cell sorting and were lysed by repeated freeze thaw cycles to release all soluble proteins of the parasite and the RBC. The peripheral, membrane-associated proteins were extracted from the pellet fraction containing the membranes by sodium carbonate buffer (pH 11). The remaining integral membrane proteins were extracted with Triton X-100. This fractionation revealed that Tex1 was partly found soluble but equal amounts of the protein could only be extracted by carbonate buffer indicating that Tex1 associated with membranes (**Figure 7**). As control for the integrity of our fractions we used monoclonal antibodies against serine-rich antigen 5 (SERA5), a soluble protein found in the PV [17], [18], [19]; MAHRP1 was used as control representing an integral membrane protein [20], [21]; MSP1 served as control representing a glycosylphosphatidylinositol lipid anchored protein on the merozoite surface and also as marker for the integral membrane fraction [22]

**Figure 8:**
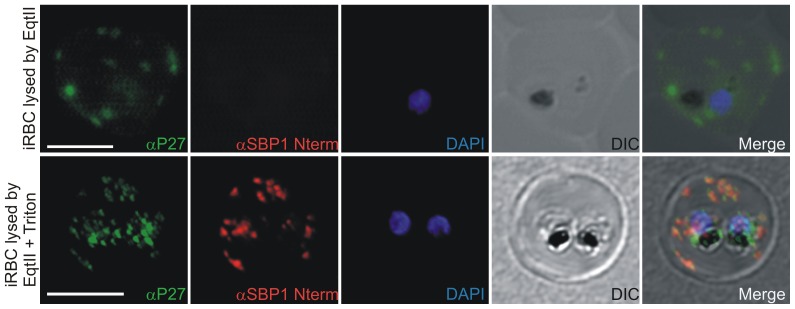
Equinatoxin II assay. A) 3D7 infected RBC lysed with equinatoxin II. Integrity of MCs is demonstrated by the absence of the SBP1 signal after using SBP1 N-terminus specific polyclonal mouse sera (note: N-terminus of SBP1 faces the lumen of MCs). Tex1 signal on the MC surface was obtained with P27-specific polyclonal rabbit sera (in green). B) 3D7 infected RBC lysed with equinatoxin followed by Triton lysis. MC lumen is now accessible for antibodies as shown by the SBP1 signal (in red). Nuclear DNA stained with DAPI (blue), Transmission image (DIC). Scale bar: 5 µm.

In order to analyze the localization of Tex1 at the MC, infected RBCs were lysed with Equinatoxin II (EqtII), a pore-forming toxin binding preferentially to sphingomyelin-containing membranes [13]. It lyses the RBC membrane ensuring integrity of PVM and MC membranes [23]. SBP1 is an integral membrane protein localizing to MCs. The C-terminus of SBP1 is directed to the RBC cytosol, whereas the N-terminus is directed to the lumen of MCs. The upper panel of **Figure 8** shows Tex1 localization in EqtII lyzed parasite infected RBC. In these EqtII treated parasites the N-terminus of SBP1 is not detected because antibodies specific to this part cannot access their target due to intact MC membranes.

SBP1 staining was performed to demonstrate the integrity of the MC membrane. P27-specific antibodies detected Tex1 in cells treated with EqtII at the MC. This demonstrated the localization of Tex1 at the surface of MC facing the RBC cytosol (**Figure 8**).

In the lower panel of **Figure 8**, the parasites were further lyzed with Triton X-100, which permeabilized also the MCs membrane, therefore SBP1 can be detected with antibodies directed against the N-terminus of the protein (**Figure 8**).

### Export of Tex1 is Brefeldin A sensitive

**Figure 9:**
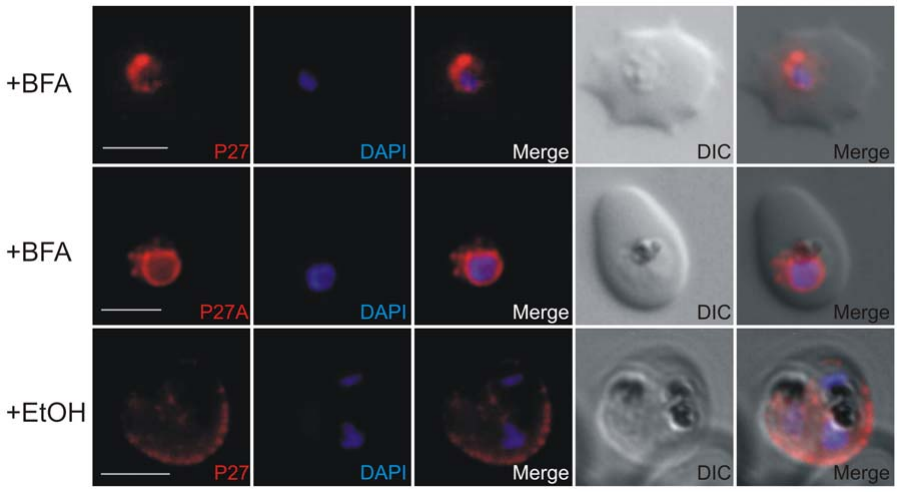
Brefeldin A sensitivity of Tex1 export. 3D7 infected RBC were treated with BFA and fixed (+BFA). Tex1 was stained using P27 or P27A-specific mouse antibodies (in red, upper panel: early trophozoite, middle panel: trophozoite). Tex1 visible inside the parasite in close proximity to the nucleus. A control culture (+ETOH) was incubated with equivalent concentration of ethanol, the solvent of Brefeldin A. In the control culture Tex1 was correctly exported and associated to MC (in red). The nucleus was stained with DAPI (in blue). Transmission image (DIC). scale bar: 5 µm.

Protein secretion pathways in the eukaryotic cell are classified into the classical and nonclassical secretory pathway as reviewed by [24]. Whereas the classical secretory pathway involves co-translational translocation of proteins into the ER or posttranslational insertion into the ER followed by vesicular transport from the ER via Golgi to the cell surface or the extracellular space reviewed in [25], the molecular mechanisms involved in the nonclassical protein secretion are independent of the ER/Golgi system [26], [27]. To test by which route Tex1 is exported, infected RBCs were treated with Brefeldin A (BFA), a fungal metabolite shown to block the classical protein secretion pathway [28]. BFA treatment blocked Tex1 export (**Figure 9**) suggesting that Tex1 export depends on components of the classical secretory pathway.

## Discussion

Extensive preclinical evaluation of the annotated hypothetical protein Tex1 revealed that two regions, the intrinsically unstructured region P27A and the coiled coil domain P27, show great potential as new malaria vaccine candidates [1], [3], [5]. Its clinical development, currently in phase 1, called for an in depth analysis of the cytological characteristics of Tex1, which was named “Trophozoite exported protein 1” due to its localization to MC at the trophozoite stage. Association to MC was confirmed by co-localization with Rex1 and other MC proteins. Tex1 associated with the MC membranes facing the cytosol of the RBC. This was demonstrated by EqtII lysis of infected RBCs, which in contrast to Triton X-100 permeabilizes exclusively the RBC membrane. While antibodies detected Tex1, other antibodies, directed against the luminal N-terminus of SBP1, could not access the lumen of MCs and thus gave no signal.

Exported proteins in *P. falciparum* are classified based on the presence or absence of the PEXEL motif which is mostly located downstream of a hydrophobic stretch. Recently, an increasing number of PEXEL-negative exported proteins (PNEPs) were identified [12], [16], [29], [30]. Tex1 also is a PEXEL negative exported protein. To date it is only poorly understood how PNEPs are trafficked across the PVM, and sequence signatures responsible for export across the PVM and to the MC remain to be identified, if these exist at all.

A common characteristic of PNEPs seems to be the presence of either N-terminal signal sequence or a transmembrane domain [31]. For Tex1 no classical signal sequence, nor PEXEL motif, could be identified. The Tex1 expression pattern varies from that of PNEPs. Whereas Tex1 is expressed in trophozoites, PNEPs are expressed early in the intra-erythrocytic developmental cycle. We identified a potential alternative start site at position - 43 in respect to the predicted translational start site (PlasmoDB, **Figure S2**). This stretch of 43 aa was predicted by SignalP (http://www.cbs.dtu.dk/services/SignalP/) to function as signal anchor and is unique for *P. falciparum* Tex1. No such preceding sequence stretch was detected in the orthologues of *P. vivax* (PVX_113335) and *P. knowlesi* (PKH_114650, **Figure S2**). Constructs of Tex1 including a GFP tag at the C-terminus were generated with or without the 43 aa hydrophobic stretch and episomally expressed. However, the GFP signals of both variants remained inside the parasite. More experimental data is needed to further investigate sequences responsible for Tex1 export. GFP-tagging of Tex1 might have interfered with the function of the RING domain at its very C-terminus. This would suggest that the RING domain plays an important role in Tex1 export. Brefeldin A treatment resulted in the accumulation of Tex1 at close proximity to the nucleus suggestive for ER or ER exit sites, indicating the involvement of the classical secretory pathway in the export of Tex1.

Tex1 exhibited a differential solubility pattern, whereby a portion of the protein was found in the soluble fraction, while the rest was present as peripheral membrane protein. No soluble Tex1 was detected in the RBC cytosol or PV, as demonstrated in the fractionation experiment using saponin lysed infected RBCs (**Figure 1B**), suggesting that the soluble pool of Tex1 is present exclusively within the parasite. This finding suggests that Tex1 changes its solubility during export: Tex1 is exported as a soluble protein, but associates with MC membranes after export. Our solubility assay showed equal amounts of soluble Tex1 and membrane-associated Tex1. However, the soluble portion likely is overrated due to freeze/thaw-mediated release of Tex1 from its MC's association.

Also for other proteins a solubility change after export has been reported, e.g. for Rex1 [32]. Similar to Tex1, Rex1 was found to associate with MCs via protein-protein interaction [32]. Rex 1 has a predicted transmembrane domain and its alpha-coiled coil region (amino acids 160–370) seems to be responsible for MC association [32]. Tex1 contains three putative coiled coil domains (**Table S3**). The alpha-helical coiled coil motif is a very abundant protein motif present in around 10% of all proteins [33]. Coiled coils have been shown to function as protein-protein interaction sites and to be involved in oligomerization and complex formation [34]. Thus, coiled coils participate in many cellular processes, such as membrane fusion, vesicular trafficking and cell motility. Further experiments are needed to elucidate the function of Tex1 coiled coil domains for MC membrane association.

Also for PfEMP1 a change in solubility during export had been reported [35]. Despite the presence of a transmembrane domain, PfEMP1 seems to be synthesized as a carbonate extractable protein. After export PfEMP1 becomes increasingly insoluble [35].

Noteworthy was the observation of very good co-localization of both peripheral membrane proteins Tex1 and Rex1, in contrast to the incomplete/partial co-localization of Tex1 with the integral membrane proteins MAHRP1 and SBP1 at MC's. This provides further evidence for a peripheral membrane association of Tex1.

We investigated, whether the export of Tex1 is influenced by other exported proteins. Tex1 export was not altered in D10 parasites (data not shown), which have a partial deletion of chromosome 9 and a truncation of chromosome 2, eliminating 22 genes, including Rex1, 2, 3, 4 and KAHRP, and resulting in loss of cytoadherence [36], [37], [38], [39] and alteration of the MC structure [40], [41]. Similarly, in MAHRP1 knock out parasites, where PfEMP1 trafficking to the RBC membrane is blocked [16], Tex1 was correctly exported and its association with the MC remained intact (data not shown).

Tex1 orthologues were found in *P. vivax* and *P. knowlesi* as well as in *P. berghei*, *P. chabaudi or P. yoelii*. P27 was highly conserved among *Plasmodium* species (**Figure S3A**). Interestingly, the unstructured region was present exclusively in *P. falciparum* (**Figure S3B**). Many of the other ring stage exported proteins of *P. falciparum,* such as MAHRP1 and 2, SBP1 and Rex1, 2, 3, and 4, as well as the *resa*-multi gene family, do not have orthologues in *P.vivax*. Discrepancies were found also in a comparison of *P. falciparum* and *P. vivax* transcription profiles [42]. Eleven percent of syntenic genes of *P. vivax and P. falciparum* differed in gene expression during the intra-erythrocytic developmental cycle [42]. Similar results were obtained for *tex1* transcripts. According to PlasmoDB the *P. vivax* orthologue showed a completely different transcriptional profile with transcripts up-regulated in ring stage parasites suggesting a divergent evolution of Tex1 function.

Antibodies directed against P27 and P27A of Tex1 were effective in *in vitro* parasite killing in the presence of monocytes [1], [3] and both P27 and P27A were recognized by serum from semi-immune adults from various endemic settings [1], [3]. These results suggested that Tex1 holds a crucial immunological function. However, we found that Tex1 was absent on the surface of the infected RBC. The effector function of Tex1-specific antibodies excludes therefore blocking of cytoadherence or opsonization and destruction of iRBC by phagocytic cells, but involves monocytes. We conclude that the activation of monocytes by P27/P27A-specific antibodies may occur after parasite egress.

The persistence of Tex1 until egress could indicate functional activity at the end of the 48 h blood stage cycle. To elucidate the biological function of Tex1, we attempted to knock-out *tex1*. These attempts failed indicating that the *tex1* locus resists recombination events due to an essential role of Tex1 for parasite survival.

## Conclusion

Tex1 was identified based of extensive preclinical evaluation as promising novel vaccine candidate against *P. falciparum* blood stage infection. In the past, malaria blood-stage vaccine development has focused on antigens located on the surfaces of iRBC or free merozoites. This approach assumed that protective antibodies would opsonize, block invasion or prevent sequestration. Tex1 was not found to be surface exposed, but instead localized to the surface of MC. Upon egress, Tex1 gets exposed to the host immune system. A Tex 1-specific antibody effector function remains to be elucidated, but likely involves the presence of monocytes.

## Supporting Information

Figure S1 **Absence of surface exposure of Tex1.** The absence of Tex1 from the surface of infected RBCs was shown by incubating live cells with P27-specific polyclonal mouse sera directed against Tex1 (panel A). Tex1 signal was detected only in a lysed cell (panel A, white arrow). Nucleus stained with DAPI (panel B). Merged pictures of both signals and the transmission image (panel C). Scale bar: 5 µm.
(TIF)

Figure S2 **Upstream region of Tex1 and its orthologues in** ***P. vivax (PVX_113335)*** **and** ***P. knowlesi (PKH_114650)*** **.** Sequence highlighted in gray represents the region upstream of the of the predicted start Methionine. Stars (*) represent stop codons.
(TIF)

Figure S3 **Sequence alignment of the** ***P. falciparum*** **Tex1 with the** ***P. vivax*** **orthologue.** A) Sequence alignment of the Tex1 C-terminus, P27 highlighted in grey. B) Sequence alignment of the Tex1 N-terminus, predicted signal sequence highlighted in light grey; P27A highlighted in bold.
(TIF)

Table S1 Oligonucleotide sequences used for cloning (restriction sites in bold).
(DOCX)

Table S2 Oligonucleotide sequences used for qRT-PCR.
(DOCX)

Table S3 Alpha-helical coiled coil domains in Tex1 (P27 in bold).
(DOC)
