# Supplementary figures and images for: Cell Biological Characterization of the Malaria Vaccine Candidate Trophozoite Exported Protein 1
Source: PLoS One. 2012 Oct 8;7(10):e46112. doi: 10.1371/journal.pone.0046112 (PMC3466242; doi:10.1371/journal.pone.0046112)

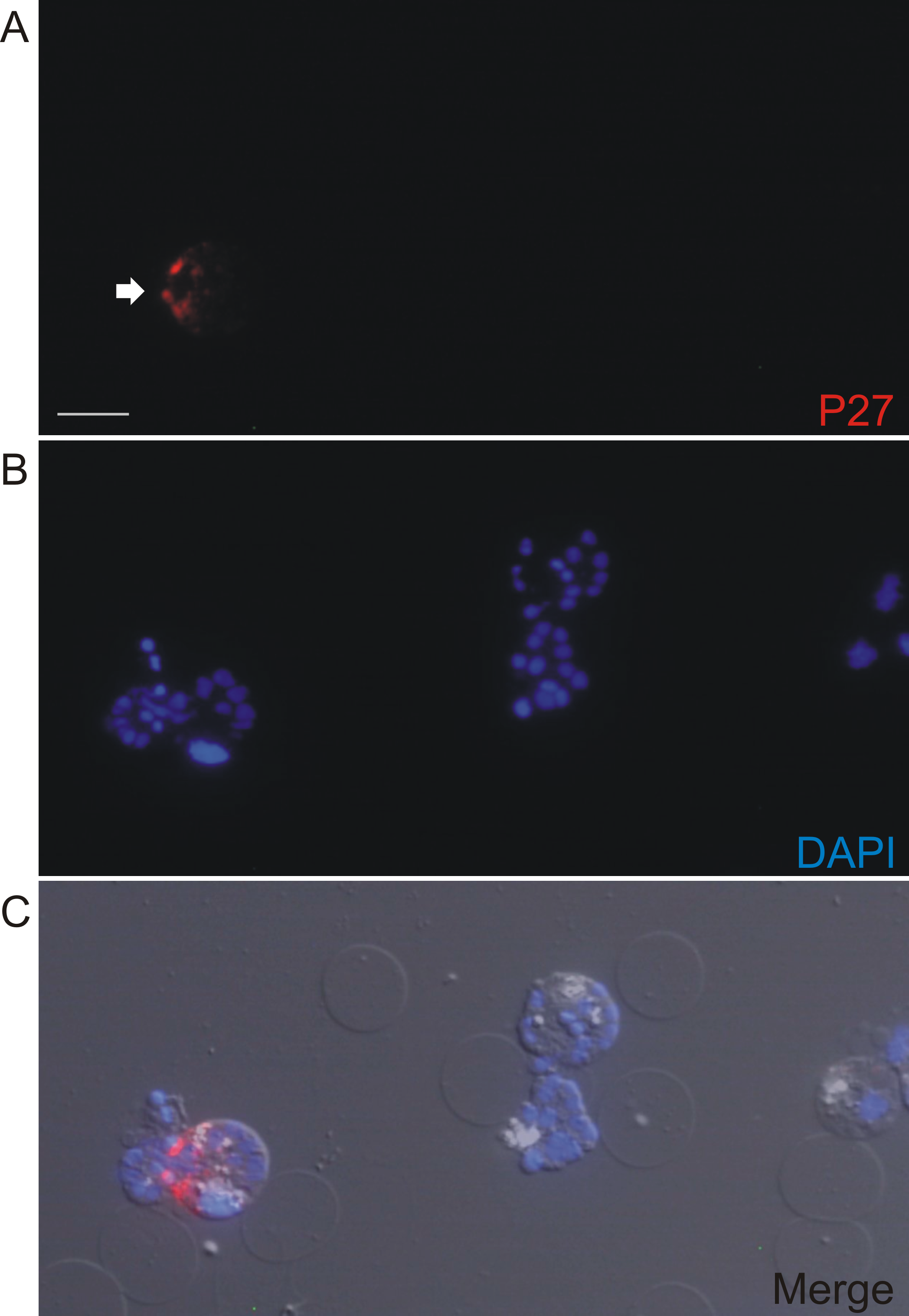

Supplement: Figure S1 — Absence of surface exposure of Tex1. The absence of Tex1 from the surface of infected RBCs was shown by incubating live cells with P27-specific polyclonal mouse sera directed against Tex1 (panel A). Tex1 signal was detected only in a lysed cell (panel A, white arrow). Nucleus stained with DAPI (panel B). Merged pictures of both signals and the transmission image (panel C). Scale bar: 5 µm. (TIF) [file pone.0046112.s001.tif]

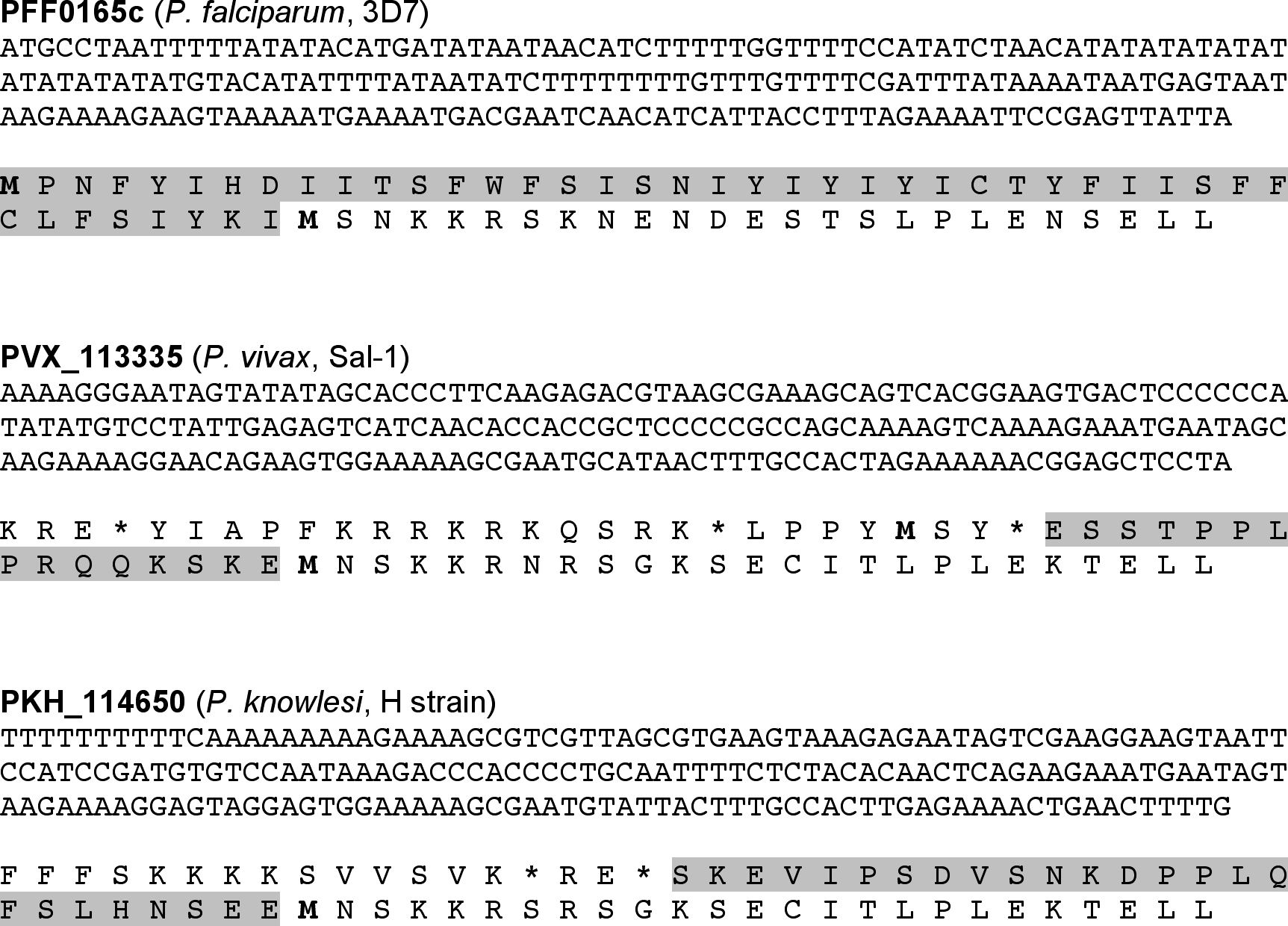

Supplement: Figure S2 — Upstream region of Tex1 and its orthologues in P. vivax (PVX_113335) and P. knowlesi (PKH_114650) . Sequence highlighted in gray represents the region upstream of the of the predicted start Methionine. Stars (*) represent stop codons. (TIF) [file pone.0046112.s002.tif]

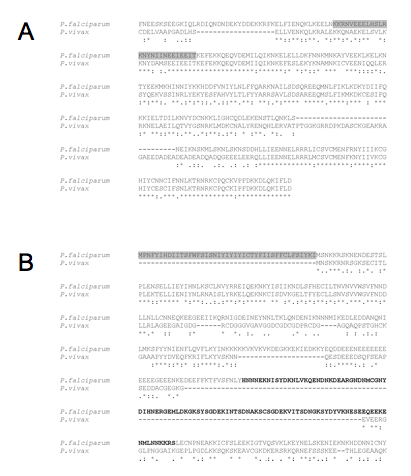

Supplement: Figure S3 — Sequence alignment of the P. falciparum Tex1 with the P. vivax orthologue. A) Sequence alignment of the Tex1 C-terminus, P27 highlighted in grey. B) Sequence alignment of the Tex1 N-terminus, predicted signal sequence highlighted in light grey; P27A highlighted in bold. (TIF) [file pone.0046112.s003.tif]
