# Supplementary material for: Cell Biological Characterization of the Malaria Vaccine Candidate Trophozoite Exported Protein 1
Source: PLoS One. 2012 Oct 8;7(10):e46112. doi: 10.1371/journal.pone.0046112 (PMC3466242; doi:10.1371/journal.pone.0046112)
Supplement: Table S1 — Oligonucleotide sequences used for cloning (restriction sites in bold). (DOCX) [file pone.0046112.s004.docx]

**Table S1: Oligonucleotide sequences used for cloning (restriction sites in bold)**
