# Supplementary material for: Cell Biological Characterization of the Malaria Vaccine Candidate Trophozoite Exported Protein 1
Source: PLoS One. 2012 Oct 8;7(10):e46112. doi: 10.1371/journal.pone.0046112 (PMC3466242; doi:10.1371/journal.pone.0046112)
Supplement: Table S3 — Alpha-helical coiled coil domains in Tex1 (P27 in bold). (DOC) [file pone.0046112.s006.doc]

**Table S3: alpha-helical coiled coil domains in Tex1 (P27 in bold)**
